# Accelerating Initiation of Adequate Antimicrobial Therapy Using Real-Time Decision Support and Microarray Testing

**DOI:** 10.1097/pq9.0000000000000191

**Published:** 2019-08-05

**Authors:** Michael J. Tchou, Heidi Andersen, Eric Robinette, Joel E. Mortensen, Eleanor A. Powell, Andrea Ankrum, Matthew C. Washam, David B. Haslam, Joshua D. Courter

**Affiliations:** From the * Department of Pediatrics, University of Colorado School of Medicine, Aurora, CO; † Section of Hospital Medicine, Children’s Hospital of Colorado, Aurora, CO; ‡ James M. Anderson Center for Health Systems Excellence, Cincinnati, OH; § Division of Infectious Diseases, Cincinnati Children’s Hospital Medical Center, Cincinnati, OH; ¶ Department of Pediatrics, University of Cincinnati, College of Medicine, Cincinnati, OH; ‖ Division of Infectious Diseases, Akron Children’s Hospital, Akron, OH; ** Department of Pathology and Laboratory Medicine, Cincinnati Children’s Hospital Medical Center, OH; †† Department of Infection Prevention and Control, Cincinnati Children’s Hospital Medical Center, Cincinnati, OH; ‡‡ Division of Infectious Diseases, Nationwide Children’s Hospital, Columbus, OH; §§ Division of Pharmacy, Cincinnati Children’s Hospital Medical Center, Cincinnati, OH, USA

## Abstract

**Introduction:**

Bloodstream infections (BSI) represent a common cause of sepsis and mortality in children. Early and adequate empirical antimicrobial therapy is a critical component of successful treatment of BSI. Rapid PCR-based diagnostic technologies, such as nucleic acid microarrays, can decrease the time needed to identify pathogens and antimicrobial resistance and have the potential to ensure patients are started on adequate antibiotics as early as possible. However, without appropriate processes to support timely and targeted interpretation of these results, these advantages may not be realized in practice.

**Methods:**

Our Antimicrobial Stewardship Program (ASP) implemented a quality improvement initiative using the Institute for Healthcare Improvement’s Model for Improvement to decrease the time between a nucleic acid microarray result for Gram-positive bacteremia and the time a patient was placed on adequate antimicrobial therapy. The primary effective intervention was a near real-time notification system to the managing physicians of inadequate antimicrobial therapy via a call from the ASP team.

**Results:**

Following the intervention, the average time to adequate antimicrobial therapy in patients with Gram-positive BSI and inadequate coverage decreased from 38 hours with the nucleic acid microarray result alone to 4.7 hours when results were combined with an ASP clinical decision support intervention, an 87% reduction.

**Conclusions:**

The positive effects of rapid-detection technologies to improve patient care are enhanced when combined with clinical decision support tools that can target inadequate antimicrobial treatments in near real time.

## INTRODUCTION

Bloodstream infections (BSI) represent a common cause of sepsis and mortality in children,^1–3^ with a high rate of morbidity and mortality in the United States.^4,5^ An important factor in achieving the best outcomes from sepsis and bacteremia is early antimicrobial therapy that properly targets the causative pathogenic organism.^6,7^ Delays in appropriate therapy lead to increased mortality and morbidity and increased length of hospitalization and healthcare costs.^8,9^ Previous pediatric quality improvement projects in sepsis demonstrate that decreasing the time to adequate antimicrobial therapy leads to improvements in care outcomes.^10,11^

Antimicrobial resistance increasingly challenges our ability to provide adequate empirical coverage in patients with BSI even when using multiple, broad-spectrum agents. The 24- to 72-hour delay inherent in traditional biological amplification techniques for pathogen identification and susceptibility testing leaves a large window for inadequate antimicrobial therapy to continue undetected. Recently developed rapid pathogen detection and identification technologies attempt to narrow that window by providing this information to clinicians up to 42 hours earlier through the use of nucleic acid microarrays.^12,13^ In practice, reductions in actual time to adequate antimicrobial agents can be improved through partnerships with antimicrobial stewardship teams^14^ and direct communication with medical staff.^15^

At Cincinnati Children’s Hospital Medical Center (CCHMC), the Verigene Gram-Positive Blood Culture (BC-GP) assay (Nanosphere Inc., Northbrook, IL) was utilized for all initial blood cultures with Gram-positive organisms starting in October, 2013. After implementation, the results were validated locally.^16^ Subsequently, the Antimicrobial Stewardship Program (ASP) determined that 1 of every 40 patients with a BC-GP result was on inadequate antimicrobial therapy. Interviews with care providers and diagnostic clinical laboratory staff indicated that despite implementing the technology, our clinical teams frequently did not act upon this new information when it was available.

Based on these observations, our ASP undertook a quality improvement initiative with a global aim of harnessing rapid pathogen identification technology to reduce adverse outcomes in patients receiving antimicrobial therapy. Adverse outcomes include effects related to both overuse and underuse of appropriate antimicrobial agents, including adverse drug reactions, prolonged infection, and promotion of resistant bacterial overgrowth. Our specific aim for this quality improvement project was to reduce the time to adequate antimicrobial therapy from 38 to 12 hours by June 1, 2016, for patients who were on inadequate antimicrobial therapy at the time of BC-GP results. We theorized that optimizing this process would reduce the time to first negative blood culture through earlier initiation of adequate antimicrobial therapy in patients with Gram-positive cocci bacteremia who were receiving inadequate empirical antimicrobial therapy.

## METHODS

### Context

CCHMC is a large, quaternary care pediatric center with >700 beds spread over 2 inpatient facilities and 16 outpatient facilities. The health system has ~33,000 admissions, 170,000 emergency and urgent care visits, and 1 million outpatient visits annually including many patients at high-risk for bacteremia due to indwelling central lines and immune compromise. The Diagnostic Infectious Diseases Testing Laboratory has an average of 57 positive blood cultures each month, with an average of 84% of these blood cultures collected from inpatients. Before our improvement project, nurses were notified of any BC-GP results and subsequently notified providers. The ASP is led by an infectious diseases physician and a clinical pharmacist with 0.5 FTE each dedicated to stewardship activities. The ASP committee is composed of multidisciplinary representatives from the divisions of Infectious Disease and Hospital Medicine as well as representatives from Infection Control, the Diagnostic Infectious Diseases Testing Laboratory, and data analyst support from the James M. Anderson Center for Health Systems Excellence. The ASP utilizes a clinical decision support system (Vigilanz Corp, Chicago, IL), which provides nearly instantaneous monitoring and notification of culture results and antimicrobial orders to facilitate automated and targeted interventions. In addition, CCHMC has a quality improvement infrastructure coordinated via the James M. Anderson Center for Health System’s Excellence which has shaped institutional culture and acceptance of improvement initiatives.

### Planning the Intervention

**Fig. 1.**
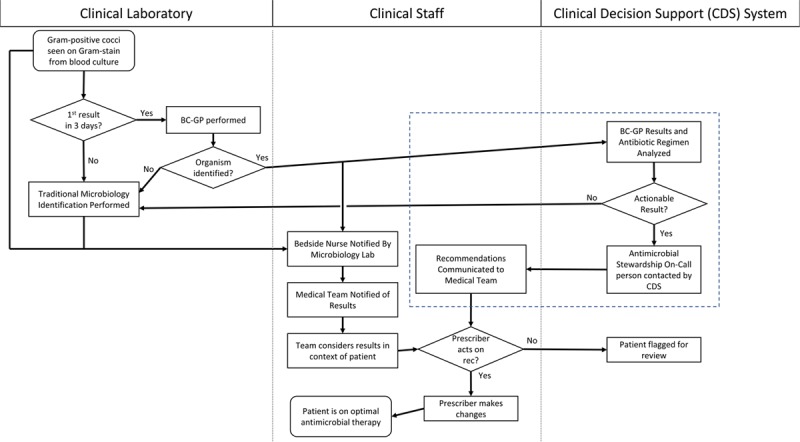
Gram-positive cocci bacteremia decision algorithm. Process map of the communication algorithm created to respond to a BC-GP result with inadequate antimicrobial coverage and rapidly notify front-line clinical providers of the need to change antimicrobial coverage. Flow diagram is organized by three clinical domains: Clinical Laboratory processes, Clinical Staff processes, and Vigilanz Clinical Decision Support processes. New process steps created through this improvement project are located within the dashed line box. Of note, the notification of bedside nurse by clinical laboratory of results was a part of standard workflow both before and after the algorithm implementation.

The ASP team used the Institute for Healthcare Improvement Model for Improvement^17^ as the framework for this QI project. First, the team evaluated the baseline scope of the problem by reviewing all cases of Gram-positive cocci bacteremia in our clinical decision support system from May 1, 2015 to April 27, 2016. The team identified patients without adequate antimicrobial therapy ordered at the time of any BC-GP result as opportunities for intervention. Team members reviewed the electronic medical records (EMR) for qualifying patient encounters and interviewed front-line providers to understand the systems failures that led to delays in changing to adequate antimicrobial therapy after BC-GP results indicated a patient was on inadequate therapy. The ASP team mapped the process of delivering BC-GP results to frontline providers to understand key decision and failure points (Fig. 1). In addition to understanding these provider-level factors, the ASP team reviewed aggregated institutional microbiologic susceptibility data to inform future interventions. A pareto chart of the initial baseline cultures was created to display the most common pathogens with inadequate antimicrobial therapy in our baseline period (see Supplemental Digital Content, available at http://links.lww.com/PQ9/A110) and baseline data were evaluated to establish the current time to adequate therapy for the study population. Finally, a review of current evidence on the risk of BSI and implementation of rapid pathogen diagnostic technology was performed.

Based on this review, the ASP team developed a clinical decision support pathway to identify and rapidly mitigate patients with BSI who were on inadequate therapy. Using evidence-informed consensus building among key stakeholders, the ASP team defined *adequate therapy* for individual pathogen and resistance genes from the BC-GP results (Table 1). These definitions were based on published spectra against wild-type organisms modified by genotypic resistance markers.^18^ A previous study at our institution had validated these genotypic markers against traditional phenotypic testing.^16^ Notably, the team categorized vancomycin as inadequate therapy for methicillin-susceptible *Staphylococcus aureus* (MSSA) based on literature indicating that a beta-lactam antimicrobial generally achieves more rapid clearance of the bloodstream via increased bactericidal activity.^19,20^ An *actionable result* was defined as any patient who was not on adequate therapy at the time a BC-GP result became available. An algorithm was created to utilize our clinical decision support tool to actively identify cases of actionable results and create an automatic notification to the designated ASP-team-member via a text message using the hospital paging system (Fig. 1). This text message was sent immediately regardless of the time of day or if it was a weekend or holiday. After notification, the ASP person-on-call would directly notify the primary care team of the inadequate antimicrobial therapy. If adequate antimicrobial therapy was not achieved after this notification, the case was flagged for review at future ASP meetings.

**Table 1.**
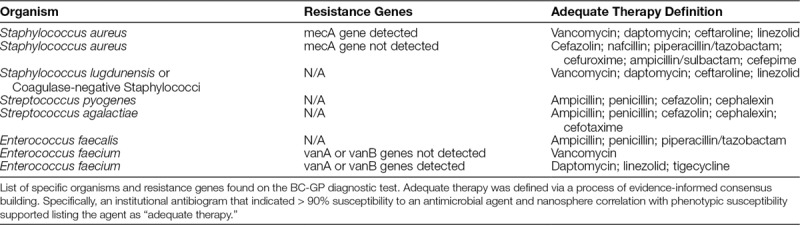
Criteria for Actionable Results

This quality improvement project was considered exempt from formal review by the Cincinnati Children’s Hospital Medical Center institutional review board.

### Improvement Activities

Improvement activities are presented below in chronological order and were implemented for the entire institution unless otherwise noted.

#### Initiating the Algorithm

To better understand any potential problems with algorithm implementation, the ASP team first implemented the algorithm for a single organism, MSSA. Automated alerts were created in the clinical decision support system. These alerts scanned all BC-GP results that were positive for MSSA, specifically a species identified as *S. aureus* and no mecA gene detected. If these patients did not have an active order for a beta-lactam with activity against MSSA, designated ASP team members were notified via a text page. No process errors were identified for this subset of BC-GP results for 3 patients over a 1-month period, so the ASP team expanded the clinical decision support algorithm to include all Gram-positive organism BC-GP results. Results were reviewed by the ASP team at weekly meetings when new data were available.

#### Ongoing Review of Patient Cases and Special Cause

After initial roll-out of the notification system, the ASP group reviewed cases and special cause variations at ongoing meetings to assess the effectiveness of the intervention and any potential gaps in the current notification process. Specifically, after a series of patient encounters for patients with actionable results that demonstrated prolonged time to adequate antimicrobials, a system of intermittent case review was established to routinely review outliers and better understand failures of the algorithm.

#### Provider Group Education

We performed 3 ramps of educational interventions. Initially, we provided just-in-time information to individual providers via a notification call from the ASP person-on call. We subsequently provided large-group presentations with the Division of Hospital Medicine and the Division of Infectious Disease.

### Measures

We defined our primary outcome measure as *time to adequate therapy*, the time difference between when a blood culture within our study population became positive to the time when an order for an adequate antimicrobial agent was placed in the electronic order entry system. To evaluate the impact of our interventions on patient-level outcomes, we also measured *time to first negative blood culture*, defined as the time difference between when a blood culture became positive and the time when the first subsequent culture was drawn which demonstrated no bacterial growth with no further positive cultures within 7 days. This measure was evaluated individually for each pathogen, as specific species had widely divergent times to negative blood cultures. As our institution does not routinely collect multiple blood cultures from children, there is some chance that growth from a blood culture may represent contamination by skin flora. One population which was excluded from the measure due to an anecdotally high false-positivity rate were patients with epidermolysis bullosa.

### Analysis

Measures were evaluated using statistical process control charts and run charts,^21,22^ and the Western Electric rules for determining special cause variation were employed. Time to adequate antimicrobial therapy and time to first negative blood culture were evaluated using an XMR control chart.^21^

## RESULTS

**Fig. 2.**
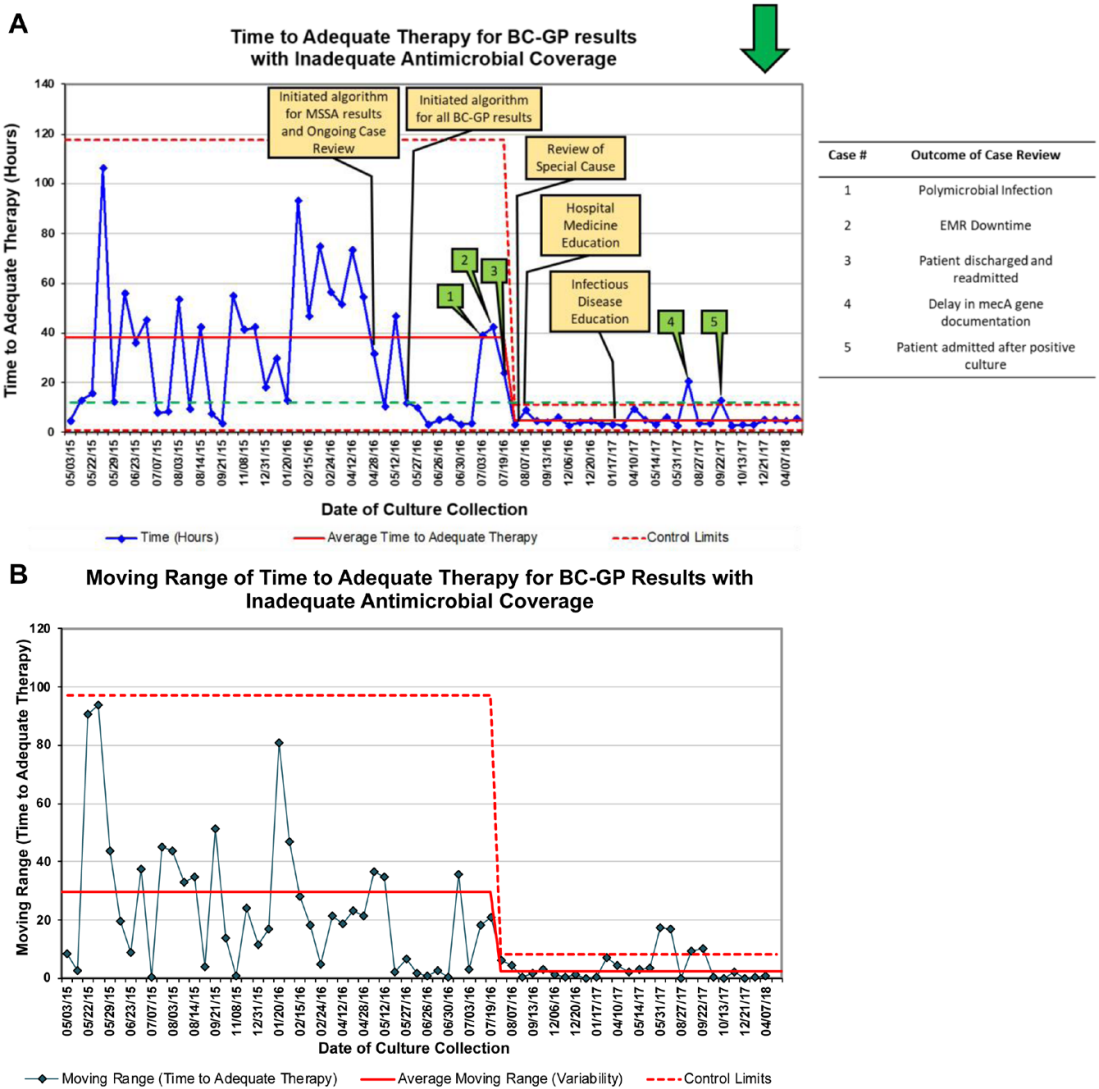
Time to adequate therapy. (A), Statistical process control X-chart displaying the time to adequate therapy and (B) moving range chart displaying the point-to-point variation in time to adequate therapy. Green arrow in upper right-hand corner indicates desired direction of change of the measure. Yellow boxes indicate PDSA interventions. Green boxes indicate the results of specific case reviews detailed in the table to the right. After sustaining the intervention, both the time to adequate therapy and the variation in the time to adequate antibiotic therapy decreased significantly.

Over the course of 28 baseline cultures, the mean time to adequate antimicrobial therapy was 38 hours (Fig. 2). Following the initiation of our clinical decision support algorithm interventions, we saw a sustained reduction of mean time to adequate antimicrobial therapy to 4.7 hours and a significant reduction in the variation of time to adequate antimicrobial therapy (Fig. 2). In general, time to adequate antimicrobial therapy declined dramatically after our algorithm was rolled out, with several notable exceptions. Early after our algorithm was introduced, 3 eligible patient encounters demonstrated increased time to adequate therapy. Case reviews of these encounters identified unique patient-specific factors that led to delayed initiation of adequate antimicrobials. These patient factors included (1) a patient with a polymicrobial infection where an Infectious Diseases consult team recommended continuation of combination therapy that did not meet the technical definitions for adequate therapy in our algorithm, (2) an EMR downtime that led to a delay in our algorithm notification of the ASP person-on-call, and (3) a patient who was identified with bacteremia after being discharged leading to a delay to initiation of therapy due to travel time back to the hospital. Following the shift in the mean time to adequate antimicrobial therapy, 2 other patient encounters were identified as special cause related to patient-specific factors during this time period. Specific organisms identified by BC-GP throughout the project are found on Table 2.

**Table 2.**
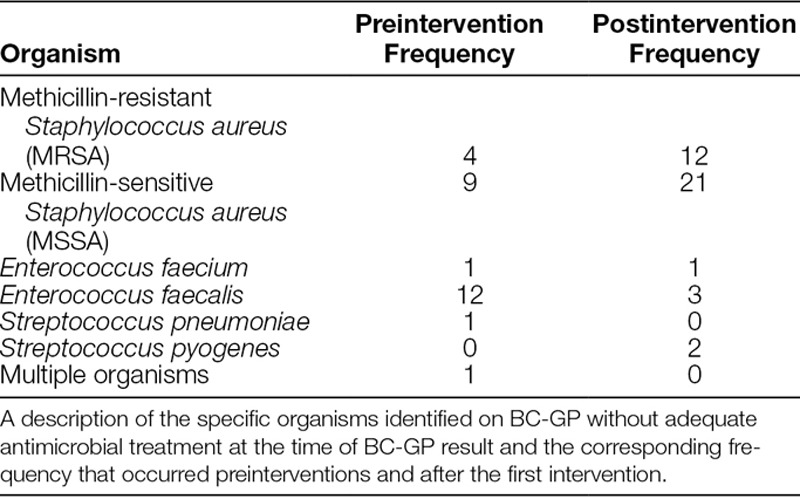
Description of Organisms Flagged by Algorithm

**Fig. 3.**
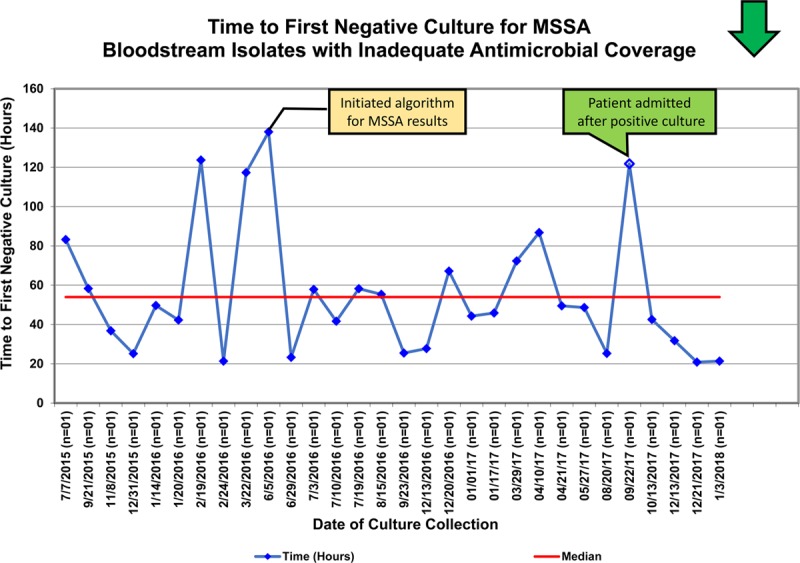
Time to first-negative culture for MSSA with inadequate antimicrobial coverage. A run chart displaying the time to first negative blood culture after a positive culture for MSSA. Yellow box indicates PDSA intervention specifically related to the MSSA population. Green box indicates the results of specific case review.

**Fig. 4.**
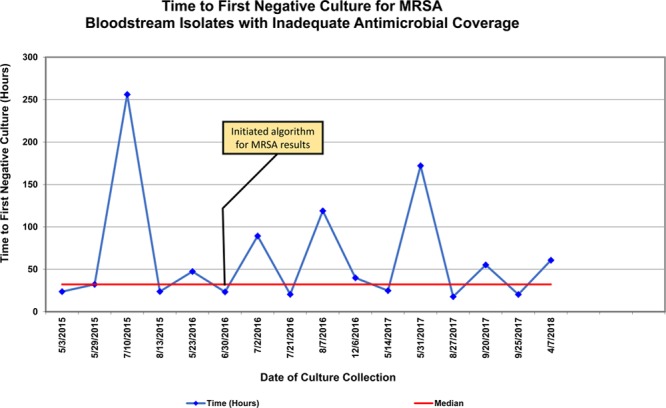
Time to first-negative culture for MRSA with inadequate antimicrobial coverage. A run-chart displaying the time to first negative blood culture after a positive culture for MRSA. Yellow box indicates PDSA intervention specifically related to the MRSA population. After initiation of the algorithm, no clear change in the time to first negative culture or variation was seen with blood cultures growing MRSA.

For our secondary measure, time to first negative blood culture, only MSSA and MRSA had a sufficient sample size to evaluate trends via run charts. For MSSA, chart review determined that 1 patient who was admitted after an outpatient blood culture became positive led to a prolonged time before the first dose of adequate antibiotics. Outside of this encounter, there was a notable decrease in the variation in time to first negative blood culture displayed on the run chart compared with the baseline period. (Fig. 3). There was no observed shift in the centerline for the time to first negative blood culture for MSSA (54 hours). For MRSA, there was no observable special cause variation in the median time to first negative blood culture (32 hours) (Fig. 4).

## DISCUSSION

The use of improvement science methods, rapid bacterial identification technology, and a real-time decision support tool supported by ASP reduced the time to adequate antimicrobial therapy by 33 hours, an 87% reduction from baseline. In addition, the encounter-to-encounter variation in the time to adequate therapy was also significantly reduced. Previous studies have shown significant reductions in the time to organism identification and susceptibility reporting using rapid diagnostic technologies as well as decreased time to adequate antibiotic therapy.^23–25^ Our project succeeded in nearly eliminating this time lag between the test result and the clinical response. Our initial experience demonstrated that implementation of the laboratory test alone did not effectively impact clinical outcomes. Targeted clinical decision support with appropriate supporting education was required to translate the potential of the new test into meaningful patient outcomes. This result is consistent with a recent systematic review indicating that the use of rapid diagnostic technology may have minimal effect without support and education from antimicrobial stewardship teams.^26^ In particular, the real-time notification of front-line clinicians that is integrated into our algorithm may provide significant advantages over other reporting algorithms which rely on specific non-real time mechanisms, such as a consultation with an infectious disease physician or phone call notifications that only occur during the daytime hours.

In addition, our study suggests that consistent earlier initiation of adequate antimicrobial agents could be associated with earlier clearance of organisms from the blood and reduced variation in this clearance time. The time to first negative culture for MSSA measure demonstrated no special cause variation, but demonstrated decreased variation and potentially an early trend towards a modest decrease in the time to first negative culture in this patient population. Because this is a rare event and a small sample size in this study, we believe that additional follow-up data will be needed to strengthen this finding. Studies of rapid diagnostic technology in adults have identified reduced length of stay and mortality related to implementation of this technology,^26^ and it is likely that earlier treatment and clearance of bacteremic organisms may be a substantial part of these improved outcomes. In pediatric populations, mortality is rare, and the complex patient populations and small sample size of this study limit our ability to measure changes in length of stay; however, we speculate that modest improvements in the time to organism clearance might improve patient outcomes. With time, further evaluation of diagnosis specific times to negative culture may allow a more granular understanding of the impact of this technology.

Use of computer-based surveillance and decision support is considered a key element to effective ASP teams.^27^ Clinical decision support, if implemented correctly, can offer great benefits to front-line clinicians.^28^ Systems that can contact appropriate providers in “real-time”, as soon as 5-minutes after a result is posted in the EMR, may offer benefits for conditions like BSI that are time sensitive. In addition, while pop-up reminders that occur in the EMR require a provider to be logged into the system, a clinical decision support system with an ability to directly communicate with providers can further reduce any delays. With broadening adoption of this technology, there is also a risk that providers can be overwhelmed with excessive notifications, in a similar manner to the widely described phenomenon of “alert fatigue.” As few as 4%–10% of medication pop-ups contain salient information that leads to changes in patient management.^29^ Our algorithm was designed to minimize this fatigue through only offering clinical decision support notifications for a targeted group of patient encounters where the benefits of a change in management are clear. This strategy of focused clinical decision support communicated in a rapid manner has the potential to offer great impact while not impeding clinical efficiency and can likely be applied to multiple clinical scenarios.

## LIMITATIONS

Our study had several important limitations. The interventions occurred at a quaternary care children’s hospital with a strong improvement science division, an active antimicrobial stewardship team, a clinical decision support program that is integrated with its EMR, and a history of multiple successful quality improvement projects, which may affect generalizability to other environments. Also, our patient population includes multiple diagnoses associated with frequent bacteremia which may affect the degree of variation displayed by the time to negative culture; however, it is unlikely to explain the sustained large reductions in time to adequate antibiotics. Retrospective-prospective data collection poses a risk of selection bias but we used objective inclusion criteria. Because there is no control group to account for secular trends, it is possible the results were affected by slow adoption of the alert system over time, rather than our interventions but the time-series nature of our data and the close temporal association of the shift with the intervention argues against this interpretation.

## NEXT STEPS

The next steps for our improvement team are to expand on the learnings from creating an algorithm for Gram-positive bacteremia and create a similar pathway for Gram-negative bacteremia. In addition, our current process involves notification of a member of the ASP team, which is another task burden with limited FTE support for our ASP. We are planning to remove this potentially unnecessary step using a notification system that integrates with Vigilanz to directly contact front-line providers through a secure messaging app, and only notifies designated ASP team members if appropriate changes to antimicrobials do not occur in a timely fashion. Finally, our current algorithm focuses on adequate antibiotics, however achieving the most appropriate therapy by reducing overuse of overly broad antibiotic coverage and reducing antibiotic use for skin contaminants is also possible through a similar mechanism and aligns with future ASP goals.

## CONCLUSIONS

We reduced the relative time to the use of adequate antimicrobial therapy by over 87% for patients with Gram-positive cocci bacteremia who are on inadequate therapy. In addition, we witnessed a reduction in variation in both the time to adequate antimicrobial therapy and the time to first negative blood culture for our patient population. These results add to the evidence that rapid-detection technologies may improve patient care by targeting interventions to patients receiving inadequate antimicrobial treatments, especially when combined with near real-time clinical decision support tools and oversight by antimicrobial stewardship teams.

## DISCLOSURE

The authors have no financial interest to declare in relation to the content of this article.

## References

[1] Watson RS Carcillo JA Linde-Zwirble WT The epidemiology of severe sepsis in children in the United States. Am J Respir Crit Care Med. 2003;167:695–701. 12433670 10.1164/rccm.200207-682OC

[2] Larru B Gong W Vendetti N Bloodstream infections in hospitalized children: epidemiology and antimicrobial susceptibilities. Pediatr Infect Dis J. 2016;35:507–510. 26766146 10.1097/INF.0000000000001057

[3] Zingg W Hopkins S Gayet-Ageron A; ECDC PPS study group. Health-care-associated infections in neonates, children, and adolescents: an analysis of paediatric data from the European centre for disease prevention and control point-prevalence survey. Lancet Infect Dis. 2017;17:381–389. 28089444 10.1016/S1473-3099(16)30517-5

[4] Balamuth F Weiss SL Neuman MI Pediatric severe sepsis in U.S. children’s hospitals. Pediatr Crit Care Med. 2014;15:798–805. 25162514 10.1097/PCC.0000000000000225 PMC4221502

[5] Kutko MC Calarco MP Flaherty MB Mortality rates in pediatric septic shock with and without multiple organ system failure. Pediatr Crit Care Med. 2003;4:333–337. 12831416 10.1097/01.PCC.0000074266.10576.9B

[6] Zasowski EJ Claeys KC Lagnf AM Time is of the essence: the impact of delayed antibiotic therapy on patient outcomes in hospital-onset enterococcal bloodstream infections. Clin Infect Dis. 2016;62:1242–1250. 26945013 10.1093/cid/ciw110 PMC4845789

[7] Natarajan G Monday L Scheer T Timely empiric antimicrobials are associated with faster microbiologic clearance in preterm neonates with late-onset bloodstream infections. Acta Paediatr. 2014; 103:e418–e423. 24990532 10.1111/apa.12734

[8] Rhodes A Evans LE Alhazzani W Surviving sepsis campaign. Crit Care Med. 2017;45(3):486–552. 28098591 10.1097/CCM.0000000000002255

[9] Hamdy RF Hsu AJ Stockmann C Epidemiology of methicillin-resistant *Staphylococcus aureus* bacteremia in children. Pediatrics. 2017;139(6):e20170183. 28562284 10.1542/peds.2017-0183 PMC5470503

[10] Jobson M Sandrof M Valeriote T Decreasing time to antibiotics in febrile patients with central lines in the emergency department. Pediatrics. 2015;135:e187–e195. 25489011 10.1542/peds.2014-1192

[11] Melendez E Bachur R Quality improvement in pediatric sepsis. Curr Opin Pediatr. 2015;27:298–302. 25944306 10.1097/MOP.0000000000000222

[12] Samuel LP Tibbetts RJ Agotesku A Evaluation of a microarray-based assay for rapid identification of Gram-positive organisms and resistance markers in positive blood cultures. J Clin Microbiol. 2013;51:1188–1192. 23363838 10.1128/JCM.02982-12 PMC3666768

[13] Beal SG Ciurca J Smith G Evaluation of the nanosphere verigene gram-positive blood culture assay with the VersaTREK blood culture system and assessment of possible impact on selected patients. J Clin Microbiol. 2013;51:3988–3992. 24048531 10.1128/JCM.01889-13 PMC3838064

[14] Box MJ Sullivan EL Ortwine KN Outcomes of rapid identification for gram-positive bacteremia in combination with antibiotic stewardship at a community-based hospital system. Pharmacotherapy. 2015;35:269–276. 25809178 10.1002/phar.1557

[15] Buehler SS Madison B Snyder SR Effectiveness of practices to increase timeliness of providing targeted therapy for inpatients with bloodstream infections: a laboratory medicine best practices systematic review and meta-analysis. Clin Microbiol Rev. 2016;29:59–103. 26598385 10.1128/CMR.00053-14 PMC4771213

[16] Beckman M Washam MC DeBurger B Reliability of the verigene system for the identification for Gram-positive bacteria and detection of antimicrobial resistance markers from children with bacteremia. Diagn Microbiol Infect Dis. 2019;93:191–195. 30477953 10.1016/j.diagmicrobio.2018.10.005

[17] Langley G Moen R Nolan K The Improvement Guide: A Practical Approach to Enhancing Organizational Performance. 2009 2nd ed San Francisco, CA: Jossey-Bass: A Wiley Imprint.

[18] Long S Pickering L Prober C Principles and Practice of Pediatric Infectious Disease. 2012 4th ed St. Louis, MO: Elsevier Churchill Livingstone.

[19] Wong D Wong T Romney M Comparative effectiveness of β-lactam versus vancomycin empiric therapy in patients with methicillin-susceptible *Staphylococcus aureus* (MSSA) bacteremia. Ann Clin Microbiol Antimicrob. 2016;15:27. 27112143 10.1186/s12941-016-0143-3 PMC4845304

[20] Wong D Wong T Romney M Comparison of outcomes in patients with methicillin-susceptible *Staphylococcus aureus* (MSSA) bacteremia who are treated with β-lactam vs vancomycin empiric therapy: a retrospective cohort study. BMC Infect Dis. 2016;16:224. 27215201 10.1186/s12879-016-1564-5 PMC4878066

[21] Brady PW Tchou MJ Ambroggio L Quality improvement feature series article 2: displaying and analyzing quality improvement data. J Pediatric Infect Dis Soc. 2018;7:100–103. 29040644 10.1093/jpids/pix077

[22] Benneyan JC Lloyd RC Plsek PE Statistical process control as a tool for research and healthcare improvement. Qual Saf Health Care. 2003;12:458–464. 14645763 10.1136/qhc.12.6.458 PMC1758030

[23] Beuving J Wolffs PF Hansen WL Impact of same-day antibiotic susceptibility testing on time to appropriate antibiotic treatment of patients with bacteraemia: a randomised controlled trial. Eur J Clin Microbiol Infect Dis. 2015;34:831–838. 25527447 10.1007/s10096-014-2299-0

[24] Eby JC Richey MM Platts-Mills JA Mathers AJ Novicoff WM Cox HL A healthcare improvement intervention combining nucleic acid microarray testing with direct physician response for management of *Staphylococcus aureus* bacteremia. Clin Infect Dis. 2018;66(1):64–71. 29020181 10.1093/cid/cix727

[25] Beal SG Thomas C Dhiman N Antibiotic utilization improvement with the manosphere verigene Gram-Positive blood culture assay. Proc (Bayl Univ Med Cent). 2015;28:139–143. 25829639 10.1080/08998280.2015.11929214 PMC4365105

[26] Timbrook TT Morton JB McConeghy KW The effect of molecular rapid diagnostic testing on clinical outcomes in bloodstream infections: a systematic review and meta-analysis. Clin Infect Dis. 2017;64:15–23. 27678085 10.1093/cid/ciw649

[27] Barlam TF Cosgrove SE Abbo LM Executive summary: implementing an antibiotic stewardship program: guidelines by the Infectious Diseases Society of America and the Society for Healthcare Epidemiology of America. Clin Infect Dis. 2016;62:1197–1202. 27118828 10.1093/cid/ciw217

[28] Utidjian L Kirkendall E Shelov E Clinical decision support in the pediatric hospital setting. Curr Treat Options Pediatr. 2015;1(1):48–58.

[29] Kirkendall ES Kouril M Minich T Analysis of electronic medication orders with large overdoses. Appl Clin Inform. 2014;5(1):25–45. 24734122 10.4338/ACI-2013-08-RA-0057 PMC3974246

